# Imiquimod (R837), a TLR7-Specific Agonist, Regulates Boar Sperm Motility via PI3K/GSK3α/β/Hexokinase Pathway

**DOI:** 10.3390/biology14091182

**Published:** 2025-09-02

**Authors:** Weijing Zhang, Adedeji O. Adetunji, Wenxian Zeng, Eslam M. Bastawy, Lingjiang Min, Nengshui Ding, Zhendong Zhu

**Affiliations:** 1 College of Animal Science and Technology, Qingdao Agricultural University, Qingdao 266109, China; zhangweijing202103@163.com (W.Z.);; 2 Department of Agriculture, University of Arkansas at Pine Bluff, Pine Bluff, AR 71601, USA; adetunjia@uapb.edu; 3 School of Biological Science and Engineering, Shaanxi University of Technology, Hanzhong 723001, China; 4 Institute for Mental and Physical Health and Clinical Translation (IMPACT), School of Medicine, Faculty of Health, Deakin University, Geelong 3216, Australia; e.ahmed@deakin.edu.au; 5 Faculty of Science, Ain Shams University, Cairo 11566, Egypt; 6 State Key Laboratory for Pig Genetic Improvement and Production Technology, Jiangxi Agricultural University, Nanchang 330045, China

**Keywords:** boar sperm, TLR7, R837, glycolysis

## Abstract

Mammalian sperm has a high metabolic activity and primarily relies on glycolysis and mitochondrial oxidative phosphorylation for energy supply. It has been reported that imiquimod (R837), a specific ligand of TLR7 protein, reduces the motility of sperm. However, the specific molecular mechanism by which TLR7 modulates boar sperm is unclear. In this study, the effect of R837, a Toll-like receptor 7 (TLR7) agonist, on boar sperm motility was investigated. Sperm samples were incubated with varying concentrations of R837 (0 to 0.8 µM) at different time points (30, 60, and 90 min). Findings reveal for the first time that TLR7 protein, a key component of the immune system’s Toll-like receptors, is predominantly localized in the middle section of the boar sperm tail, with a smaller concentration observed in the neck. Also, immunofluorescence (IF) revealed that approximately half of the boar sperm sample expressed TLR7. Furthermore, the TLR7 agonist influenced glycolytic hexokinase activity and mitochondrial function via the PI3K-GSK3β signaling pathway. It also selectively inhibited motility in the lower-layer sperm, while motility in the upper-layer sperm remained unaffected. Additionally, this study determined that incubation conditions for boar sperm with 0.2 μM R837 at 37 °C for 60 min yielded the most pronounced inhibition of forward motility in the lower layer of sperm, without compromising the integrity of the acrosome or plasma membrane. The present study reveals the crucial role of R837 in boar sperm motility and highlights TLR7 as an important protein that regulates boar sperm energy metabolism.

## Simple Summary

The objective of this study was to investigate the impact of imiquimod (R837), a compound that targets the TLR7 protein, on the motility of boar sperm, to elucidate the underlying mechanisms and evaluate its potential application in porcine breeding. Our findings indicate that TLR7 is predominantly localized in the midpiece of the sperm tail. R837, through its interaction with TLR7, modulates sperm energy production by inhibiting glycolysis and impairing mitochondrial function, leading to a reduction in ATP levels. This results in decreased motility of less active sperm, particularly at a concentration of 0.2 μM R837 at 37 °C for 60 min, without causing structural damage to the sperm. We concluded that R837 regulates boar sperm motility via the PI3K/GSK3α/β/hexokinase pathway.

## 1. Introduction

The quality of boar sperm is of great significance to the reproductive health, reproductive efficiency, and genetic quality of boars. It is crucial to the sustainable development and economic benefits of the breeding industry [1]. While boar sperm also features the neck and tail, the head, which contains the nucleus, carries the genetic material to be delivered to the oocyte [2]. To successfully fertilize the oocyte, the sperm initiates an acrosomal reaction to effectively penetrate the female reproductive tract and avoid phagocytosis by immune cells [3]. Compared with somatic cells, sperm locomotion requires a lot of energy. ATP is the fuel used by the axonal protease in the flagella of sperm tail [4], and active protein modification (such as phosphorylation) also depends on ATP [5]. However, sperm exhibit high chromatin aggregation and stable DNA organization, unlike somatic cells [6]. Despite being described as transcriptionally inert, this unique feature may underlie the potential role of sperm in chromatin remodeling and gene regulation during and after spermatogenesis [7]. Thus, sperm motility depends on the activation or inhibition of key protein signaling pathways [8].

TLR family proteins include a group of pathogen recognition receptors involved in immune response modulation [9]. In particular, TLR7 recognizes viruses and single-stranded RNA viruses, playing an important role in initiating innate and adaptive immune responses [10]. Zhu et al. observed that in boars infected with Classical Swine Fever (CSF) virus, the expression levels of *TLR7* in macrophages significantly increased. Additionally, the TLR7 agonist R837 not only reduced the expression of TLR7, but also inhibited the replication of CSF virus [11]. Miriam et al. found that TLR7 is strongly induced during adipocyte differentiation, during which imiquimod, another TLR7 agonist, downregulates the concentration of resistin, a cysteine-rich hormone secreted from adipocytes, in adipocyte cell-culture supernatants and modulates the gene expression of the glucose transporter Glut4 [12]. Interestingly, Fujita et al. showed that 10 μg/mL of peptidoglycan, a TLR2 agonist, and 100 ng/mL of lipopolysaccharide (LPS), a TLR4 agonist, could activate the TLR pathway after incubation with sperm for 6 h [13]. Moreover, He et al. found that the motility of boar sperm was significantly reduced after incubation with 1 μg/mL LPS [14]. Umehara et al. mapped the TLR7 distribution in mouse sperm cells and found that TLR7 influences the movement of mouse sperm [15].

The immunomodulator imiquimod, also referred to as R837, is recognized as the most potent TLR7 agonist [16]. Additionally, R837 functions as an immune response modifier with antiviral effects and impacts sperm energy metabolism [17]. Ren et al. found that when TLR7/8 is activated, phosphorylated forms of glycogen synthase kinase α/β (GSK3 α/β) and nuclear factor kappa-B (NF-κB) are present, which leads to reduced mitochondrial activity and ATP levels and impaired motility in goat sperm [18]. Wen et al. demonstrated that the TLR7/8 agonist (R848) impacts mitochondrial function through the PI3K/GSK3α/β/hexokinase and PI3K/NF-κB/hexokinase signaling pathways, resulting in inhibited bovine X sperm motility [19]. While some studies have demonstrated that TLR7 is localized in the tail of mouse sperm, a key region for glycolysis, it is imperative to acknowledge that different animal species may have different energy metabolism pathways due to their microenvironmental differences [20]. Notably, there is limited information on the role of TLR7-mediated energy metabolism in the regulation of boar sperm motility. Thus, the present study aims to investigate whether and how boar sperm motility is regulated by TLR7. Sperm motility, ATP levels, mitochondrial membrane potential and energy metabolism were analyzed after sperm were treated with TLR7-specific agonist R837. It will provide insight into the mechanism by which TLR7 agonist R837 modulates boar sperm motility, thereby providing a theoretical framework for advancing research on sperm motility regulation.

## 2. Materials and Methods

### 2.1. Materials

Unless otherwise stated, all reagents and chemicals were purchased from Sigma-Aldrich, Shanghai, China. The TLR7 antibody was obtained from Bioss (#bs 66101R) [Beijing, China], and R837 was obtained from Enzo Life Sciences (ALX-420-039-M100) [Enzo Life Sciences Inc., Exeter, PA, USA].

### 2.2. Animals

Five mature and fertile Duroc boars (18 months old) were housed at the Ao’nong Boar Breeding Farm in Binzhou, Shandong Province. Gloved-hand technique was used to collect semen from each boar once per week for five weeks. Sperm motility was evaluated using computer-assisted sperm analysis (CASA), and more than 80% of the samples were used for the swim test. All animals and experimental procedures were approved by the Qingdao Agriculture University Institutional Animal Care and Use Committee (QAU1121010).

### 2.3. Boar Sperm Swim-Up Technique

The fresh boar sperm were incubated in semen sorting solution consisting of diluent I (205 mM glucose, 12.8 mM sodium citrate, 20.39 mM sodium chloride (NaCl), 5.4 mM potassium chloride (KCl), 15.01 mM sodium bicarbonate (NaHCO_3_), 3.35 mM EDTA dissolved in 200 μL deionized water + 1% BSA) and different concentrations of R837 (0, 0.05, 0.1, 0.2, 0.4, 0.8 μM) for different periods (30, 60, 90 min) at 37 °C. Subsequently, the swim-up sperm technique was employed to separate high-motility sperm from low-motility sperm. Sperm in the upper layer and lower layer were collected and counted. Then, 1 mL of semen was added to 2 mL of incubation solution, adjusting the concentration to approximately 5 × 10^7^ sperm/mL. After incubation, 1 mL of semen from the upper layer was transferred into a new centrifuge tube, and the remainder was discarded. Similarly, for the lower layer, the top 2 mL was collected and discarded, leaving 1 mL volume in the tube [15,21].

### 2.4. Analyses of Sperm Motility

The CASA system (Sperm Class Analyzer® CASA System-Microptic, Barcelona, Spain) was used to assess the total and progressive motility of the sperm. Briefly, 6 µL of semen from the sample was collected and loaded onto a microscope slide for analysis. A total of five fields were randomly selected for evaluating motility patterns, with a minimum of 200 sperm in every field. Total motility was defined as the percentage of motile sperm moving with a path velocity greater than 12 µm/s. Progressive motility was defined as the percentage of motile sperm moving with a path velocity of 60 µm/s and maintaining a straight line for over 80% of their movement.

### 2.5. Analyses of Swim-Up Sperm Count

First, a 10 μL sperm sample was added to the edge of the cover glass and allowed to filter through the disposable full counting chamber of the hemocytometer. After standing for 5 min, the hemocytometer was placed under an inverted fluorescence microscope (ZEISS, Axio Observer 5, Oberkochen, Germany). The number of sperm in the 25 × 16 (1 mm long and 10 μm deep)-type square counting chamber was observed under a 40× microscope. The sperm count was conducted in the four corners and the middle squares with 3–5 repetitions, and the average value was calculated.

### 2.6. Analyses of Sperm Acrosome Integrity and Membrane Integrity

Upper-layer and lower-layer sperm were incubated in different solutions with R837 (0, 0.05, 0.1, 0.2, 0.4, 0.8 μM) for 30 min at 37 °C. Firstly, the membrane integrity of sperm was analyzed by the LIVE/DEAD™ Sperm Viability Kit (Invitrogen™, Shanghai, China). Briefly, 120 μL (>10^7^) of semen was incubated with 0.1 μL of SYBR-14 working solution (100 nM) and 0.5 μL of PI stock solution (2.4 mM) for 10 min at 37 °C in the dark. Five fields were randomly selected for each slide, with at least 100 spermatozoa per field, and evaluated using an epifluorescence microscope (ZEISS DM200LED, Oberkochen, Germany).

Subsequently, acrosome integrity was assessed using fluorescein isothiocyanate–peanut agglutinin (FITC-PNA) (Sigma-Aldrich, Shanghai, China). Briefly, 30 μL of sperm sample was smeared on a microscope slide to air dry. Slides were incubated with 100 μg/mL FITC-PNA solution for 30 min in a dark, moist chamber at 37 °C, followed by nuclear staining with 2.4 mM PI stock solution for 10 min. After staining, slides were fixed in 75% methanol for 10 min, and stained sperm samples were observed and photographed using an epifluorescence microscope. Five fields were randomly observed (a minimum of 100 sperm per field) for evaluation.

### 2.7. Analyses of Sperm ATP Content

ATP levels of sperm were assessed with the ATP Assay Kit (A095-1-1, Nanjing Jiancheng Bioengineering Institute, Nanjing, China) according to the manufacturer’s instructions. All reagents were thawed in advance and kept on ice before use. Semen samples (10^7^ sperm) were resuspended with assay lysate and boiled for 10 min. Then, the standard solution, sperm sample, and working solution I to working solution VI were successively added to the different groups. Finally, 200 µL was drawn from the 800 µL solution system and added to a 96-well plate. Thereafter, the sperm samples were incubated at room temperature for 5 min, and the absorbance was measured at 636 nm using a multi-detector microplate reader (TECAN, Infinite M Nano, Männedorf, Switzerland).

### 2.8. Metabolite Analysis

Metabolomics profiling was analyzed using a UPLC-ESI-Q-Orbitrap-MS system (UHPLC, Shimadzu Nexera X2 LC-30AD, Shimadzu, Kyoto, Japan) coupled with Q-Exactive Plus (Thermo Scientific, San Jose, CA, USA). The raw MS data were processed using MS-DIAL for peak alignment, retention time correction, and peak area extraction. Metabolites were identified by accurate mass (mass tolerance < 10 ppm) and MS/MS data (mass tolerance < 0.02 Da), which were matched with HMDB, MassBank, and other public databases, as well as our self-built metabolite standard library. In the extracted-ion features, only the variables having more than 50% nonzero measurement values in at least one group were kept.

### 2.9. Analyses of Sperm Mitochondrial Membrane Potential

Sperm mitochondrial status was detected using the JC-1 (lipophilic cation 5,5′,6,6′-tetrachloro-1,1′,3,3′-tetraethyl-benzimidazolylcarbocyanine iodide) Mitochondrial Membrane Potential Detection Kit (C2006, Beyotime Institute of Biotechnology, Shanghai, China). In this assay, aggregates in the mitochondrial matrix exhibit red fluorescence under high mitochondrial membrane potential (MMP) and green fluorescence under low MMP. Following the manufacturer’s instructions, 100 μL of semen was collected and centrifuged at 600× *g* for 3 min. The sperm precipitate was then resuspended in BTS diluent containing 0.3 μM R837 and incubated at 37 °C for 1 h. Subsequently, 1 mL from the upper layer and lower layer was transferred into separate tubes. A 70 μL sperm sample from the sorted semen was stained with 70 μL of 1×JC-1. After incubation at 37 °C for 20 min in the dark, the sperm were fully stained with the working solution, washed twice with JC-1 buffer, and centrifuged at 600× *g* for 3 min at 4 °C. Finally, 300 μL of JC-1 buffer was added to resuspend the sperm (5 × 10^6^), and the mitochondrial membrane potential of the sperm from the upper layer and lower layer was detected by flow cytometry (FACSAria III, BD Biosciences, Franklin Lakes, NJ, USA).

### 2.10. Analyses of Sperm Hexokinase (HK), 6-Phosphofructokinase (PFK), and Pyruvate Kinase (PK) Activities

The HK assay kit (A077-3, Nanjing Jiancheng Bioengineering Institute, Nanjing, China) was used to detect the HK activity. Briefly, after adding the extraction solution, sperm samples were centrifuged at 8000× *g* at 4 °C for 10 min, then crushed (20 kHz, 200 W, operating at 20%, 30 cycles for 3 s on and 10 s off) on ice. Then, the 30 μL supernatant was added to a mixture of six different working solutions. The absorbance of HK (20 s and 5 min 20 s) was measured with a microplate reader (TECAN, Infinite M Nano, Männedorf, Switzerland) at 340 nm. Moreover, the 6-PFK assay kit (A129-1-1, Nanjing Jiancheng Bioengineering Institute, Nanjing, China) and PK assay kit (BC0540, Solarbio, Beijing, China) were used to test the activity of PFK and PK, respectively, according to the manufacturer’s instructions. Ultimately, the absorbance of PFK (20 s and 10 min 20 s) and PK (20 s and 2 min 20 s) was measured with a microplate reader (TECAN, Infinite M Nano, Männedorf, Switzerland) at 340 nm.

### 2.11. Flow Cytometric Analysis of TLR7

First, 1 mL of semen was centrifuged at 700× *g* for 2 min, and the resulting sperm pellets were resuspended in 1 × PBS, fixed with 300 μL methanol, washed three times with PBS, and permeated with 300 μL PBST. After another hour-long wash with PBS, the sample was blocked with 10% goat serum in PBST for 30 min and incubated with a primary antibody at 1:200 at 4 °C overnight. The PBS diluted secondary antibody prepared by 1%BSA (1:200) was then added and incubated at 37 °C for 30 min in the dark. After three PBS washes, each for 5 min, the fluorescence intensity of TLR7 was detected by flow cytometry (FACSAria III, BD Biosciences, Franklin Lakes, NJ, USA). A total of 20,000 sperm events were analyzed.

### 2.12. Western Blotting Analysis

A sperm sample was lysed with RIPA buffer to extract sperm total protein, and sodium dodecyl sulfate (SDS) was added to denature the protein. The total protein (20 µg) from each sample was then separated on a 10% SDS-PAGE gel (PG112, Epizyme, Shanghai, China) and transferred onto a polyvinylidene fluoride (PVDF) membrane. The non-specific bindings of PVDF membranes were blocked with 5% (*m*/*v*) bovine serum albumin diluted with TBST (1% TBS, 0.1% Tween 20). These membranes were then immunoblotted with primary antibodies, diluted in 1% (*m*/*v*) bovine serum albumin in TBST and incubated at 4 °C for 12 h, including anti-TLR7 (#bs 6601R, Bioss, 1:1000, Beijing, China), anti-GSK3β (#bs 0023R, Bioss, 1:1000, Beijing, China), anti-phospho-GSK3β (Ser21) (#bs 5368R, Bioss, 1:300, Beijing, China), anti-PI3K (AF3241, Affinity Biosciences, 1:1000, Beijing, China), anti-phospho-PI3K p85 α (Tyr607) (AF6241, Affinity Biosciences, 1:1000, Beijing, China), and anti-α-tubulin (AC008, 1:1000, AB clonal, Wuhan, China). Subsequently, these membranes were washed 3 times with TBST, followed by incubation with horseradish peroxidase-conjugated secondary antibodies (AS014, 1:1000, AB clonal, Wuhan, China) for 2 h, and then washed with TBST thrice. Signal detection was performed using ECL plus (E412-01, Epizyme, Shanghai, China), and images were developed with a gel imaging analyzer (Alpha, Fluor Chem Q, Shanghai, China). Bands on the blots were quantified by Image J software version 1.51j8 (Media Cybernetics, Rockville, MD, USA).

### 2.13. Immunofluorescence (IF) of Sperm

Sperm samples were placed on special glass slides and washed with PBS, air-dried, and permeabilized using TritonX-100/PBS (0.1% *v*/*v*) for 1 h. Subsequently, the sperm sample was blocked with 10% goat serum (*v*/*v*) at room temperature for 30 min, followed by incubation with TLR7 primary antibody monoclonal (#bs 6601R, Bioss, 1:1000, Beijing, China) at 4 °C overnight. Sperm samples were washed with 1 × PBS thrice (5 min each time), and the antigens were visualized by Cy3-conjugated goat anti-rabbit IgG (1:150; Beyotime Institute of Biotechnology, Haimen, China, A0516) in the dark. Then, 1 μg/mL DAPI (2-(4-Amidinophenyl)-6-indolecarbamidine dihydrochloride, 1:1000; Beyotime Institute of Biotechnology, C1002) was used to stain sperm nuclear contents. The fluorescence microscope (ZEISS, DM200LED, Oberkochen, Germany) was used for sperm photography and observation. Sperm samples were incubated without TLR7 primary antibody as the negative control.

### 2.14. Statistical Analysis

All results except the metabolome data were expressed as the mean ± standard error of the mean (SEM). Before statistical analysis, all data were tested for normal distribution and homogeneity of variance. Statistical analyses of data from at least three replicates were compared using either Student’s *t*-test or one-way ANOVA, and multiple comparisons were performed with the Statistical Package for the Social Sciences (SPSS Statistics v26.0; IBM, Inc., Amunc, NY, USA). The metabolome data were analyzed using R (version 4.0.3), with R packages employed for multivariate data analyses and modeling. Data were mean-centered using Pareto scaling. Models were built on principal component analysis (PCA). Metabolites with Variable Importance for the Projection (VIP) values greater than 1.0 and *p* value less than 0.05 were considered to be statistically significant metabolites (*, *p* < 0.05; **, *p* < 0.01; ***, *p* < 0.001).

## 3. Results

### 3.1. TLR7 Expression in Boar Sperm

**Figure 1:**
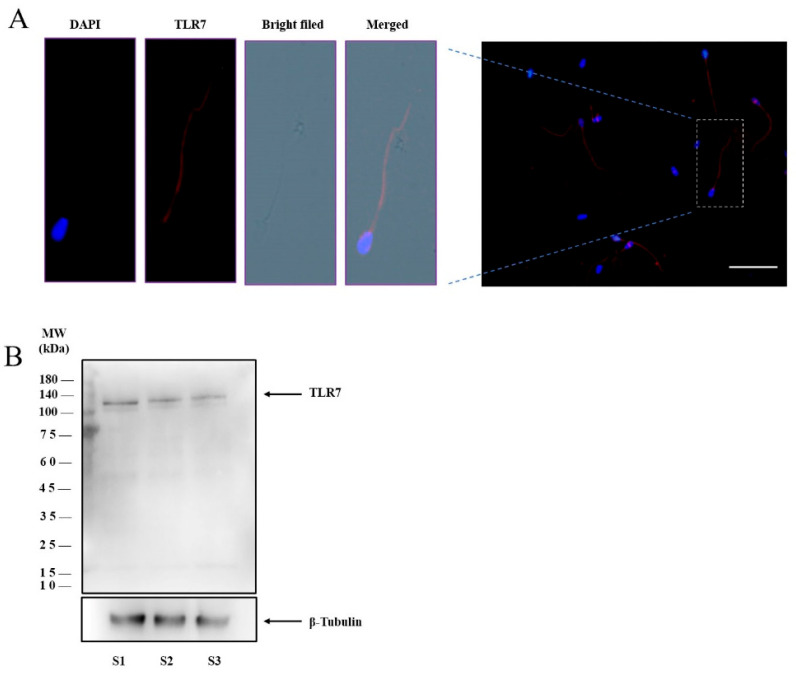
The expression of TLR7 in boar sperm. (**A**) Localization and quantification of TLR7 (red) and DAPI (blue) in boar sperm. Sperm fluorescence scale bar = 50 μm. (**B**) Detection of TLR7 proteins in boar sperm by Western blotting.

TLR7 expression was analyzed in boar spermatozoa by Western blot and immunofluorescence methods. Results show that TLR7 is localized in the sperm tail (Figure 1A), while the negative control shows no signal. Also, TLR7 protein was observed at a molecular weight of 121 kDa in boar sperm (Figure 1B; Figure S1).

### 3.2. Sperm Incubated with R837 Exhibited Decreased Motility and Preserved Acrosome and Membrane Integrity

**Figure 2:**
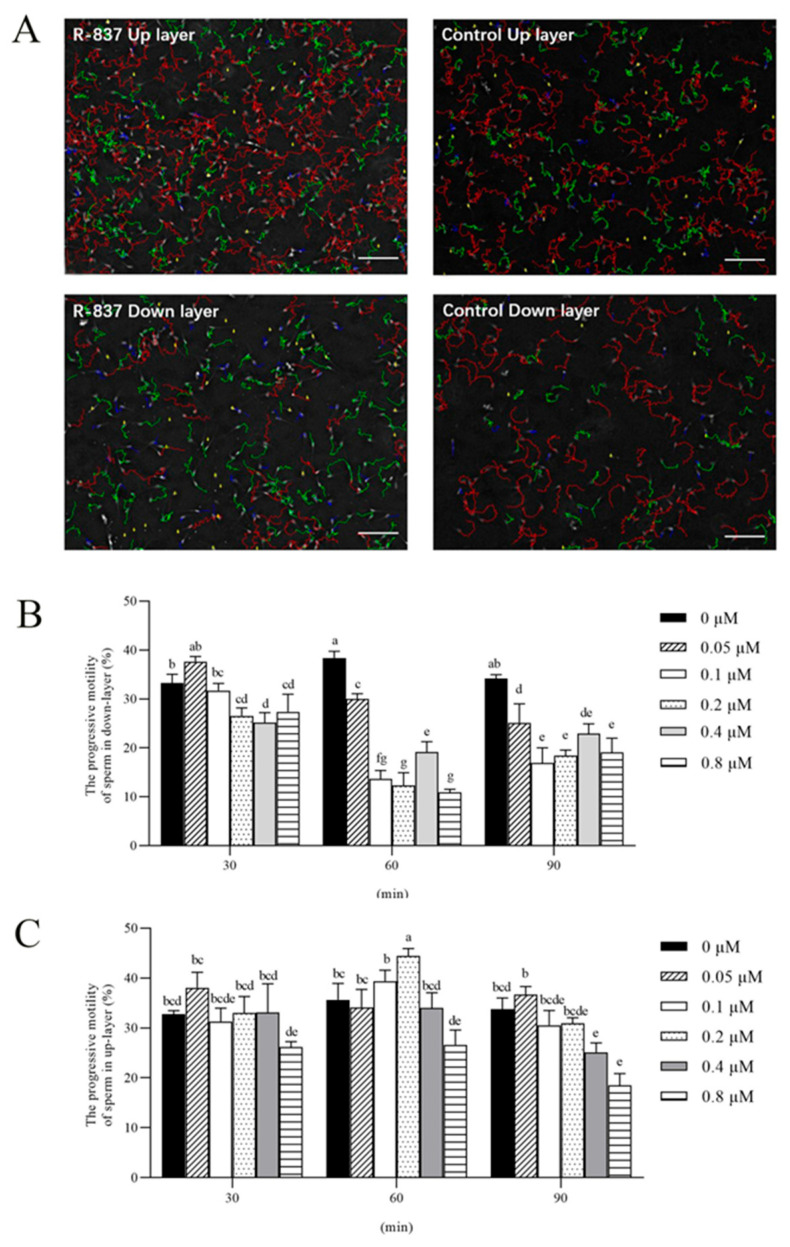
(**A**) Trajectories of upper-layer and lower-layer sperm incubated with 0.2 μM R837 for 1 h were measured by the CASA system. The red track indicates the progressive sperm (VAP > 60 μm/s), the green track indicates the moderate sperm (60 μm/s ≥ VAP ≥ 20 μm/s), and the blue track indicates the slow sperm (VAP < 20 μm/s). R-837 upper layer: The upper layer of sperm incubated with R837. Control upper layer: The upper layer of sperm not incubated with R837. R-837 lower layer: The lower layer of sperm incubated with R837. Control lower layer: The lower layer of sperm not incubated with R837. (**B**) Assessment of progressive motility of upper-layer sperm with different concentrations in 30, 60, 90 min. (**C**) Assessment of progressive motility of lower-layer sperm with different concentrations in 30, 60, 90 min. Values are means ± standard error of the mean (SEM) of 3 replicates. Scale bar = 100 μm. Different lowercase letters indicate significant differences (*p* < 0.05). VAP: average path velocity; CASA: computer-assisted sperm analysis.

**Figure 3:**
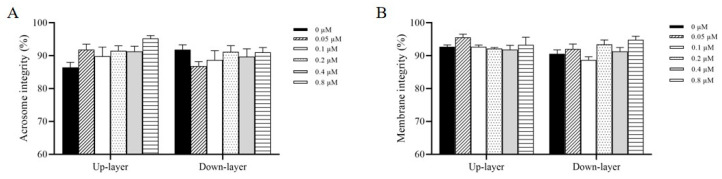
(**A**) Effects of R837 supplementation at 0.05 to 0.8 μM in diluent I for 1 h on acrosome integrity (**A**) and plasma membrane integrity (**B**) of upper layer and lower layer of sperm and membrane integrity. Values are specified as mean ± standard error of the mean (SEM) of 5 replicates. Values without different lowercase letters do not differ significantly (*p* > 0.05).

Figure 2A shows sperm movement trajectories after incubation with 0.2 μM R837 for 60 min; lower-layer sperm exhibited a shift from fast progressive movement (red trajectory) to slow progressive movement (green trajectory). Sperm incubation with R837 significantly decreased the lower-layer sperm progressive motility (PM) in a concentration-dependent manner, with reductions observed from 0.05 μM to 0.8 μM at time points of 30, 60, and 90 min (Figure 2B). Interestingly, the maximum inhibition of upper-layer sperm PM was observed in 0.2 μM R837 treatment after incubation for 60 min (Figure 2B). For the upper-layer sperm PM, it was observed that only the 0.4 and 0.8 μM R837 treatments significantly decreased (*p* < 0.05) the value of sperm PM when compared to the control after incubation for 90 min (Figure 2C). Furthermore, as shown in Table 1, lower-layer sperm motility parameters, examined by CASA, such as average path velocity (VAP), straight-line velocity (VSL), and curvilinear velocity (VCL), were lower in the R837 treatments than in the control group. To further investigate the effect of R837 on sperm acrosome and membrane status, we also evaluated the integrity of the sperm acrosome and membrane. As shown in Figure 3A,B, the sperm acrosome integrity and plasma membrane integrity in both the upper layer and lower layer did not significantly change (*p* > 0.05) when sperm were incubated with different concentrations of R837, ranging from 0.05 μM to 0.8 μM, for 60 min.

### 3.3. R837, a TRL7 Agonist, Decreased Sperm Motility Through Mitochondrial Activity and the PI3K/GSK3α/β/Hexokinase Pathway

**Figure 4:**
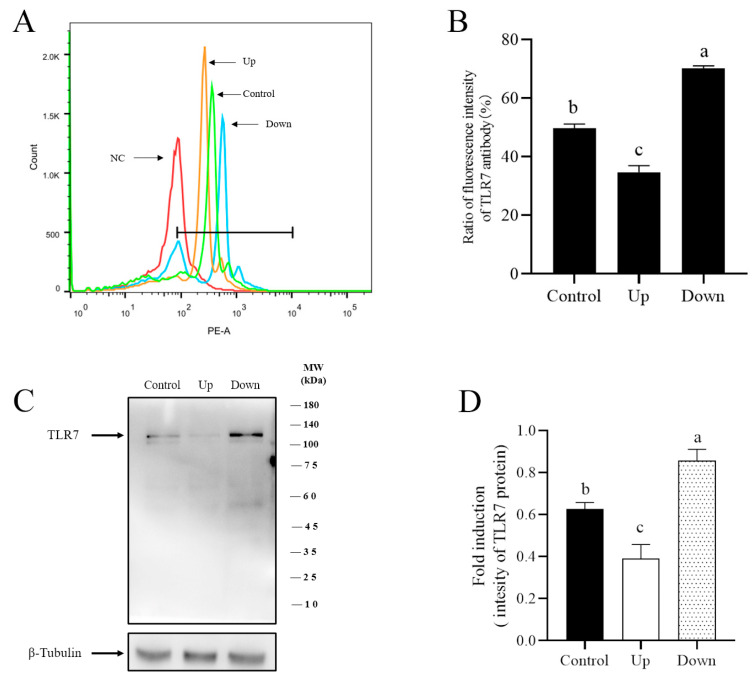
Detection of TLR7 content in the upper layer and lower layer of boar sperm by FCM and WB. (**A**,**B**) The histogram of TLR7 expression was drawn based on flow cytometer detection. (**C**) The quantitative expression of TLR7 proteins divided by α-tubulin (internal control). (**D**) Gray analysis. Values are specified as mean ± standard error of the mean (SEM) of 4 replicates. Different lowercase letters indicate significant differences (*p* < 0.05).

**Figure 5:**
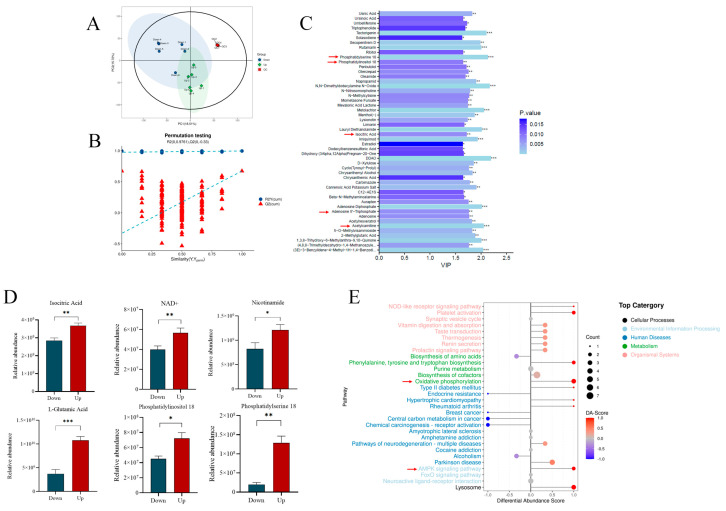
Screening and differential function analysis of upper- and lower-layer sperm metabolites. (**A**) Principal component analysis (PCA) diagrams of metabolomics analysis of upper- and lower-layer sperm cells. (**B**) OPLS-DA diagrams. (**C**) Upper- and lower-layer sperm differ in the top fifty metabolites. (**D**) Histogram analysis showed that R837 led to upregulation or downregulation of metabolites associated with the tricarboxylic acid cycle and some amino acids in lower-layer sperm. (**E**) DA score pathway analysis showed that oxidative phosphorylation and AMPK energy metabolism pathway were affected by R837. * indicates *p* < 0.05, ** indicates *p* < 0.01, *** indicates *p* < 0.001.

According to IF and flow cytometry, the expression level of TLR7 was higher in lower-layer sperm than in upper-layer sperm (Figure 4A,B). Similar results were obtained by Western blot detection (Figure 4C,D; Figure S2). In addition, given the prior result indicating that R837 impairs boar sperm motility, the metabolites associated with sperm energy metabolism were detected. Under using the LC/MS technique, 110 differential metabolites were detected between the upper and lower layers of boar sperm, including 76 downregulated and 34 upregulated metabolites. The PCA and OPLS-DA map showed differences in metabolites between the upper-layer and lower-layer sperm groups (Figure 5A–C); the metabolic pathway of the lower-layer sperm was significantly inhibited. Histograms indicated that metabolites involved in sperm ATP generation such as glutamate, phosphatidylserine, phosphatidylinositol, adenosine, isocitrate, and NAD+ were significantly downregulated in the lower-layer sperm group (Figure 5D). The differential abundance score (DA score) diagram showed that TLR7 protein regulated porcine sperm metabolism through mitochondrial oxidative phosphorylation and other pathways (Figure 5E).

**Figure 6:**
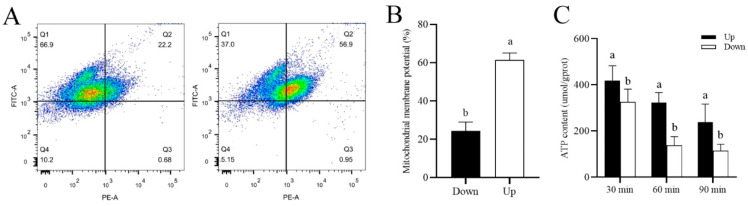
Evaluation of ATP levels and mitochondrial activity of sperm after incubation with R837. (**A**). Measurement of mitochondrial membrane potential in upper-layer and lower-layer sperm after incubation with 0.2 μM R837 for 60 min. (**B**). Mitochondrial membrane potential histogram of upper- and lower-layer sperm. (**C**). Measurement of ATP level in upper-layer and lower-layer spermatozoa after incubation with 0.2 μM R837 at different time points. Values are specified as mean ± standard error of the mean (SEM) of 3 replicates. Different lowercase letters indicate significant differences (*p* < 0.05).

As shown in Figure 6C, ATP levels in the lower-layer sperm were significantly decreased (*p* < 0.001) during incubation with 0.2 μM R837 for 90 min. In addition, the mitochondrial membrane potential of the lower-layer sperm was also significantly lower (*p* < 0.05) than that of the upper-layer sperm after adding 0.2 μM of R837 to the incubation solution for 60 min (Figure 6A,B).

**Figure 7:**
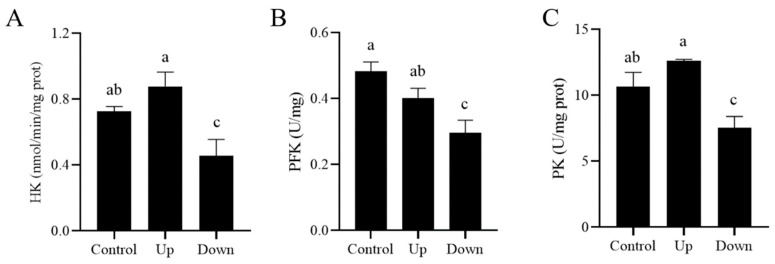
Effects of R837 supplementation at 0.05 to 0.8 μM in BTS for 1 h on rate-limiting enzymes in the glycolytic pathway: (**A**) HK activity, (**B**) PFK activity, (**C**) PK activity. Values are specified as mean ± standard error of the mean (SEM) of 3 replicates. Different lowercase letters indicate significant differences (*p* < 0.05).

**Figure 8:**
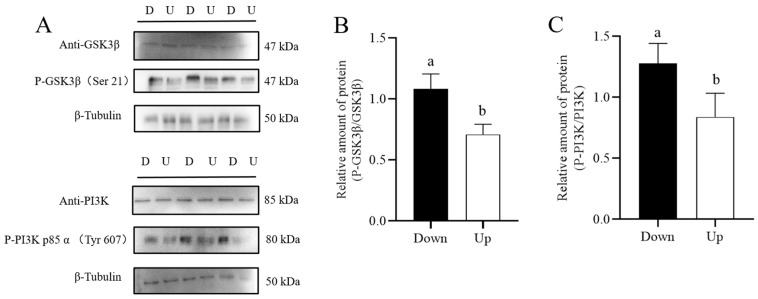
Detection of phosphorylation-related proteins in boar sperm by Western blotting. (**A**) The intensity of the bands was analyzed using a Gel-Pro Analyzer (Media Cybernetics, Rockville, MD, USA). The quantitative expression of P-GSK3β proteins over anti-GSK3β and P-PI3K proteins over anti-PI3K (**B**,C). Values are presented as means ± standard error of the mean (SEM) of 3 replicates. Different lowercase letters indicate significant differences (*p* < 0.05). D: lower-layer sperm sample; U: upper-layer sperm samples.

Meanwhile, to verify if the glycolytic pathway was impacted, the activity of HK, PFK, and PK, three glycolytic rate-limiting enzymes, was measured. We found that the TLR7 agonist R837 significantly suppressed those enzymes’ activities in the lower-layer sperm (*p* < 0.05) (Figure 7A–C). As the TLR7 agonist R837 reduced sperm ATP level and mitochondrial membrane potential in lower-layer sperm, PI3K and GSK3β, involved in the regulation of sperm energy metabolism, were also analyzed in this study. Results showed that the phosphorylation levels of PI3K and GSK3β in lower-layer sperm were significantly increased (*p*< 0.05) compared with those in the upper-layer sperm (Figure 8, Figure S3).

## 4. Discussion

The sperm motility metabolism and regulation mechanism is a complex biological process, involving a variety of cell biology and biochemistry factors, and sperm motility is affected by many factors, such as mitochondrial function, ATP metabolism, hormone level, intracellular calcium ion level, temperature, and pH [21,22,23]. Mitochondria are the primary sites for aerobic respiration of most cells and are involved in energy synthesis [24]. Thus, changes in the normal functioning of the mitochondria will result in deleterious effects on cellular function. Research has shown that Toll-like receptors (TLRs) are expressed in mammalian sperm and play a crucial role in sperm maturation and motility within the epididymis [25,26]. Interestingly, R837 can obtain R848 activity by adding a hydroxyl group and an ethoxy methyl chain to the modification [27]; hence, it is used as an immune system activator.

In this study, the boar sperm was incubated with different concentrations of R837 for various durations, namely 30, 60, and 90 min, to understand its effect on boar sperm motility. Our findings indicated that although there was no significant difference in sperm count between the upper and lower layers (Figure 2), motility in the lower layer was significantly inhibited following 60 min of incubation with 0.2 μM R837. In addition, the sperm count in the upper layer decreased significantly after 90 min, along with a significant reduction in motility, possibly due to sperm precipitation during prolonged incubation. This observation aligns with Beigi et al.’s finding, which suggests that incubating sperm samples for over 90 min adversely affects sperm biological parameters [28]. Furthermore, the CASA data demonstrated that VSL, VAP, PM, and TM of the lower layer of sperm were significantly reduced after a 60 min incubation with 0.2 μM R837 (Table 1). In contrast, no significant changes were observed in the upper layer. Given that R837 is commonly used for its immunomodulatory and anti-inflammatory activities [29], we investigated whether decreased sperm activity was due to cytotoxic effects. Our examination of sperm’s acrosome and plasma membrane integrity showed no significant differences (Figure 3), leading us to hypothesize that TLR7 influences sperm motility.

TLR7 affects cellular activity by modulating transcription and translation processes in cancer cells and immune cells. However, sperm have highly compacted chromatin and are structurally different from somatic or germ cells. These features suggest that mature sperm are generally transcriptionally and translationally inert once matured [30,31]. Therefore, TLR7 signaling does not affect sperm motility through the activation of mRNA transcription. Moreover, since TLR7 is a highly conserved transmembrane protein consisting of extracellular, intracellular, and transmembrane regions [32], we hypothesize that the extracellular region binds to specific ligands such as R837, while the intracellular region forms complexes with downstream splice molecules to transmit signals. Meanwhile, our findings indicate a significant reduction in the ATP content, mitochondrial activity, and key glycolytic enzymes of the lower-layer sperm. On this note, we further explored other signaling pathways through which TLR7 might affect sperm motility.

MyD88 is considered a key adaptor of TLR7 protein in all TLR signal transduction pathways, playing a role independent of Toll-like receptor 3 (TLR3) signaling [33]. The activation of the TLR signaling pathway is mainly stimulated by the TIR domain (Toll/IL-1 receptor) and MyD88 receptor. MyD88 attracts IL-1 receptor-associated kinase-4 (IRAK-4) to TLRs. IRAK-1 is activated by phosphorylation and interacts with TRAF6, thereby activating the IKK complex and leading to the activation of phosphatidylinositol 3 kinase (PI3K), nuclear transcription factor-κB (NFκB), mitogen-activated protein kinase (MAPK), and other signaling pathways [34]. PI3K is an intracellular enzyme that catalyzes the phosphorylation of membrane inositol lipids and plays a crucial role in various biological processes, including cell growth, differentiation, survival, and motility [35]. Previous studies have shown that activating PI3K signaling enhances sperm motility [36]. Glycogen synthase kinase 3 (GSK3) is a serine–threonine protein kinase found in the cytoplasm of all eukaryotes, and it is divided into α and β subtypes, which have similar domains and overlapping functions [37]. The α subtype regulates glycogen synthase and transcription factors such as c-Jun. In contrast, the β subtype is a constituent mitochondrial protein and a phosphorylation target of protein kinase B (AKT) [38]. In this study, we found that when sperm was cultured with R837, the phosphorylation levels of PI3K and GSK3β in the lower layer of sperm increased. PI3K is activated mainly through interaction with proteins with phosphorylated tyrosine residues, conformational changes, self-phosphorylation, or direct binding to Ras and P110 [39]. Additionally, we demonstrated that PI3K activation, caused by phosphorylation at the tyrosine 607 site, resulted in reduced sperm motility, indicating that PI3K plays a key role in sperm energy metabolism. After the activation of PI3K, PIP3, as a second messenger, binds to the pH domain of AKT, translocating AKT from the cytoplasm to the cell membrane, thus phosphorylating and activating the conformational change. The activated AKT further activates downstream GSK3 [40].

In the present study, it was observed that the TLR7 protein was expressed in the boar sperm tail, which is different from a previous study that declared TLR7 was located in the acrosome of boar sperm head [41]. The difference may be due to the different conditions of sperm permeabilized and blocking. It is noticed that there was a lack of a negative control in the immunofluorescence study performed by Wu et al. [41]. Usually, the boar sperm acrosome is detected with an unspecific signal when boar sperm immunofluorescence is performed. There is a need for further studies about the function of TLR7 on boar sperm.

## 5. Conclusions

**Figure 9:**
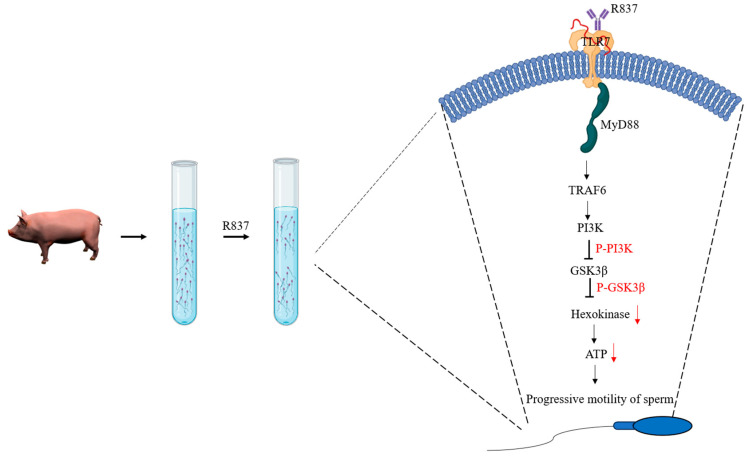
The TLR7 signal transduction mechanism affects ATP production in boar sperm. In the lower-layer sperm, the TLR7 agonist R837 inhibits hexokinase activity and reduces ATP content by phosphorylation of PI3K and GSK3 proteins, thus leading to lower-layer sperm exhibiting low motility.

In summary, as shown in Figure 9, the present study showed that R837, a TLR7 agonist, affects boar sperm mitochondrial function and glycolytic hexokinase activity through the PI3K/GSK3β signaling pathway, reducing ATP content and thereby inhibiting the motility of lower-layer sperm. Furthermore, we demonstrated that TLR7 is localized in the middle of the sperm tail and a small portion in the neck. Importantly, our findings show that incubating boar sperm with 0.2 μM R837 at 37 °C for 60 min notably inhibits the forward motility of lower-layer sperm without affecting upper-layer sperm motility. This specific concentration and incubation time also do not compromise the integrity of the acrosome or plasma membrane, making this study a key reference for subsequent applications. This study reveals the crucial role of R837 in sperm motility and highlights TLR7 as an important protein that regulates sperm energy metabolism.

## Figures and Tables

**Table 1:** Effect of R837 on boar upper-layer and lower-layer sperm motility parameters measured with CASA.

Layer	Sperm Parameter	0 μM	0.05 μM	0.1 μM	0.2 μM	0.4 μM	**0.8 μM**
Upper layer	TM (%)	86.9 ± 3.0 ^b^	92.3 ± 1.3 ^a^	89.5 ± 2.5 ^ab^	89.9 ± 1.0 ^ab^	91.3 ± 1.6 ^a^	81.6 ± 2.8 ^c^
	PM (%)	68.2 ± 3.3 ^c^	67.3 ± 2.5 ^c^	71.2 ± 1.1 ^b^	81.1 ± 1.0 ^a^	67.2 ± 3.7 ^c^	58.2 ± 3.0 ^d^
	VCL (μm/s)	70.5 ± 5.5 ^b^	73.2 ± 2.5 ^ab^	72.2 ± 1.7 ^ab^	75.3 ± 3.0 ^a^	73.3 ± 5.6 ^ab^	66.8 ± 3.4 ^c^
	VSL (μm/s)	30.0 ± 1.0	28.3 ± 1.2	30.1 ± 0.7	32.8 ± 0.6	27.7 ± 1.1	27.0 ± 0.6
	VAP (μm/s)	45.1 ± 2.7	42.1 ± 1.8	44.3 ± 1.0	49.3 ± 1.5	43.5 ± 3.5	40.3 ± 1.9
	LIN (μm/s)	43.0 ± 2.6	38.9 ± 1.2	42.0 ± 1.2	43.8 ± 2.3	38.2 ± 2.0	40.7 ± 2.2
	STR (%)	66.9 ± 3.1	67.4 ± 2.6	68.5 ± 1.2	66.7 ± 2.7	64.5 ± 3.1	67.3 ± 3.2
	WOB (%)	64.2 ± 1.2	57.8 ± 1.8	61.3 ± 0.8	65.6 ± 0.7	59.3 ± 1.9	60.4 ± 0.5
	ALH (μm)	4.5 ± 0.3	4.7 ± 0.3	4.2 ± 0.1	3.9 ± 0.1	4.6 ± 0.3	4.1 ± 0.1
	BCF (Hz)	5.1 ± 0.1	5.0 ± 0.2	5.8 ± 0.1	6.3 ± 0.3	5.6 ± 0.5	5.9 ± 0.4
Lower layer	TM (%)	85.2 ± 0.4 ^b^	90.9 ± 3.7 ^a^	89.0 ± 0.4 ^a^	87.8 ± 0.8 ^ab^	85.1 ± 1.7 ^b^	80.7 ± 0.3 ^c^
	PM (%)	76.1 ± 0.7 ^a^	74.0 ± 2.8 ^a^	54.7 ± 1.8 ^c^	53.5 ± 0.5 ^c^	61.9 ± 1.9 ^b^	57.7 ± 0.4 ^b^
	VCL (μm/s)	72.2 ± 2.0 ^a^	72.2 ± 0.8 ^a^	58.2 ± 1.6 ^c^	58.2 ± 2.4 ^c^	64.8 ± 2.5 ^b^	63.3 ± 0.1 ^b^
	VSL (μm/s)	29.4 ± 0.5 ^a^	28.5 ± 0.4 ^a^	24.0 ± 0.5 ^b^	23.8 ± 0.7 ^c^	25.1 ± 2.5 ^b^	24.2 ± 0.2 ^b^
	VAP (μm/s)	43.2 ± 1.3 ^a^	40.8 ± 0.5 ^a^	32.4 ± 1.1 ^b^	33.0 ± 1.7 ^b^	35.5 ± 1.1 ^ab^	33.5 ± 0.2 ^b^
	LIN (μm/s)	40.8 ± 0.6	39.5 ± 0.8	41.3 ± 0.3	40.9 ± 0.6	38.9 ± 1.4	38.2 ± 0.2
	STR (%)	68.2 ± 1.1	70.0 ± 1.6	74.2 ± 0.9	72.4 ± 1.8	70.9 ± 1.7	72.0 ± 0.1
	WOB (%)	59.8 ± 0.2	56.4 ± 0.3	55.6 ± 0.3	56.5 ± 0.7	54.8 ± 0.8	53.0 ± 0.3
	ALH (μm)	4.2 ± 0.1	4.2 ± 0.2	3.6 ± 0.1	3.6 ± 0.1	3.8 ± 0.1	3.9 ± 0.1
	BCF (Hz)	6.1 ± 0.1	6.4 ± 0.8	6.6 ± 0.1	6.5 ± 0.2	7.5 ± 0.3	5.8 ± 0.1

Values are expressed as mean ± standard deviation. Different letters within a line indicate a significant difference (*p* < 0.05). VCL, curvilinear velocity; VSL, straight-line velocity; VAP, average path velocity; BCF, beat-cross frequency; ALH, lateral head; STR, straightness (VSL/VAP); LIN, linearity (VSL/VCL); WOB, wobble (VAP/VCL), *n* = 5. Different lowercase letters differ significantly (*p* < 0.05).

## Data Availability

The original contributions presented in this study are included in the article/Supplementary Materials. Further inquiries can be directed to the corresponding authors.

## Supplementary Materials

The following supporting information can be downloaded at: https://www.mdpi.com/article/10.3390/biology14091182/s1, Figure S1: WB original images of Figure 1; Figure S2: WB original images of Figure 4; Figure S3: WB original images of Figure 8.
