# Supplementary material for: Imiquimod (R837), a TLR7-Specific Agonist, Regulates Boar Sperm Motility via PI3K/GSK3α/β/Hexokinase Pathway
Source: Biology (Basel). 2025 Sep 2;14(9):1182. doi: 10.3390/biology14091182 (PMC12467471; doi:10.3390/biology14091182)

Figure S1. WB original images of Figure 1.

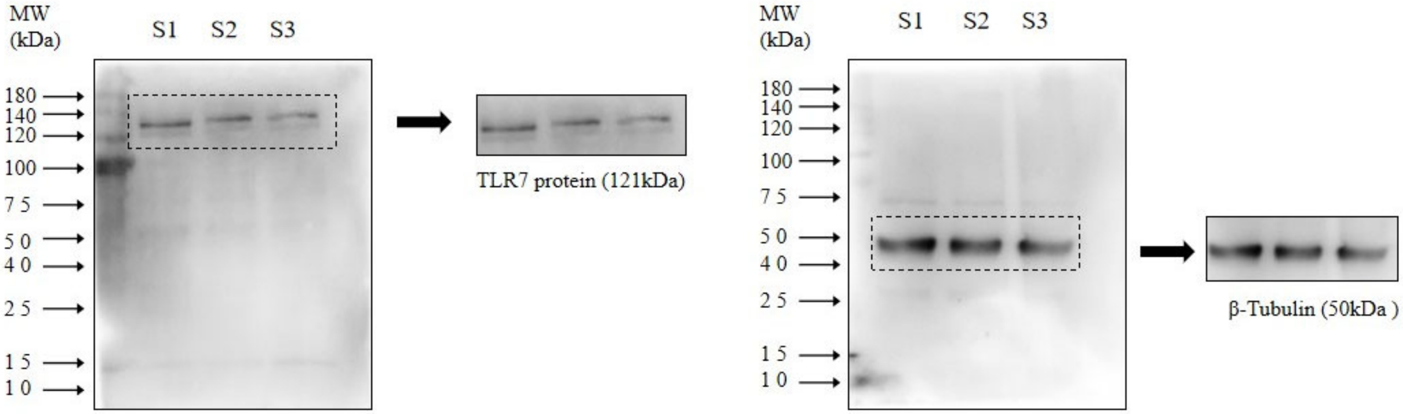

Figure S2. WB original images of Figure 4.

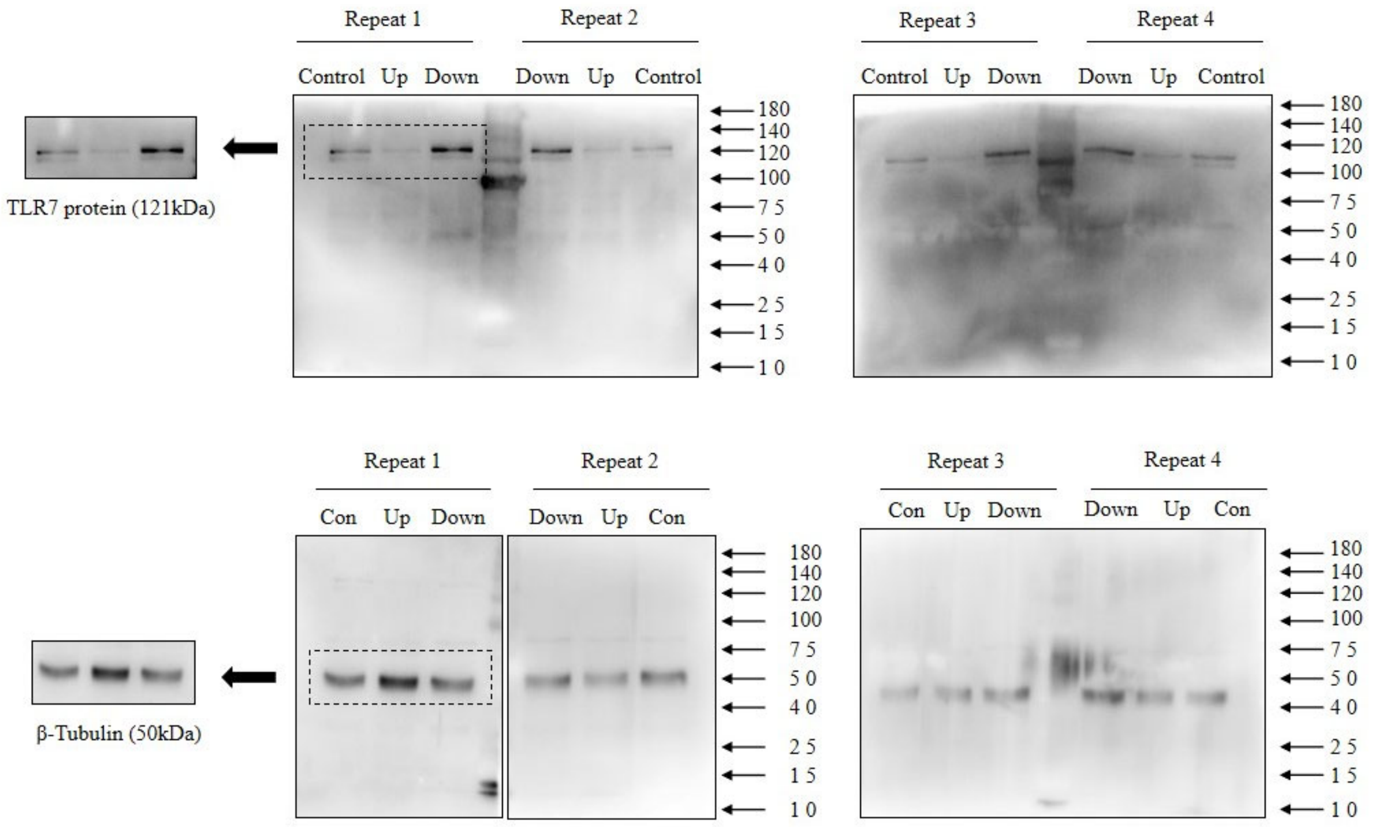

Figure S3. WB original images of Figure 8.

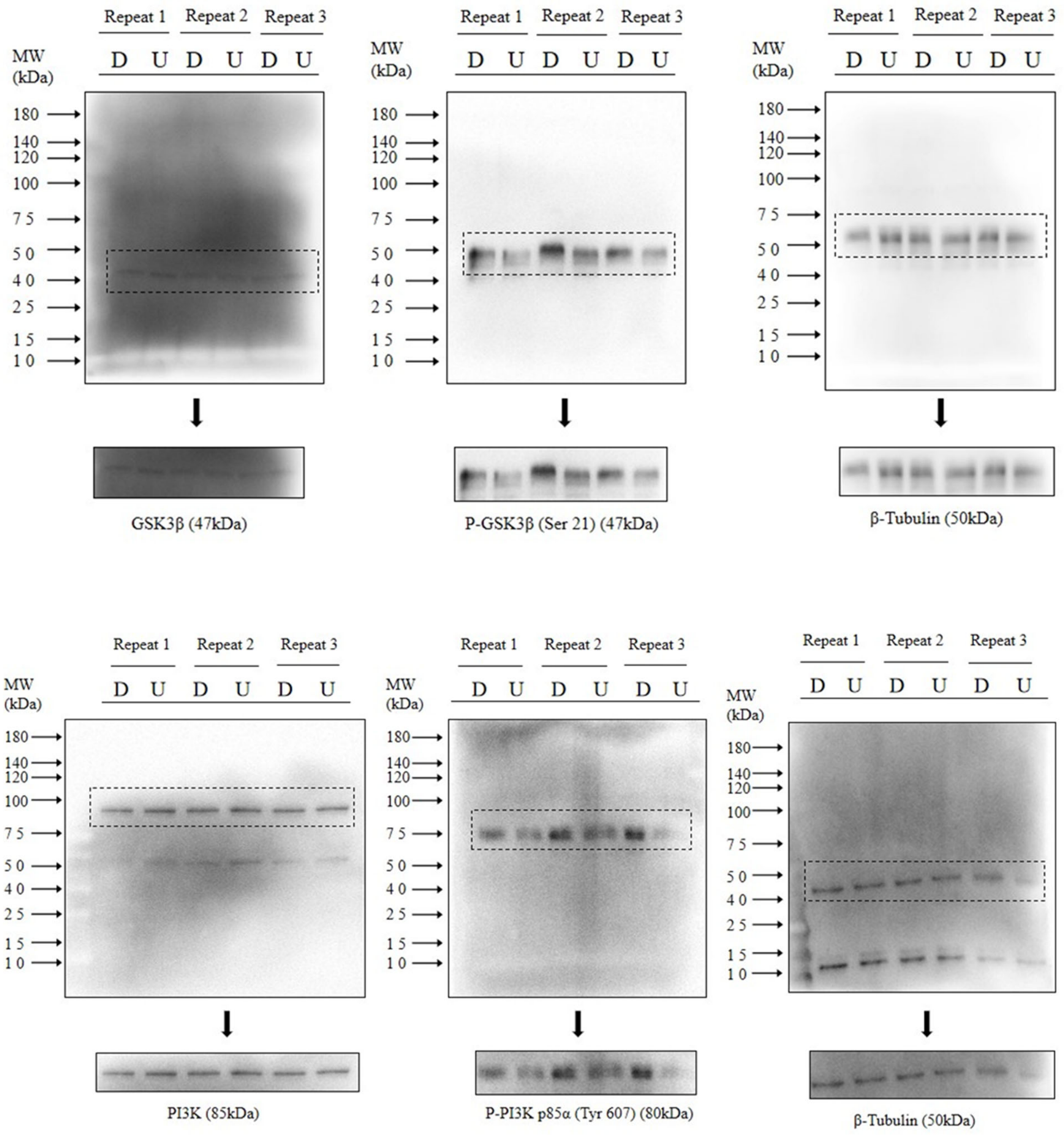

Supplement: Supplementary file 1 [file biology-14-01182-s001.zip › biology-3746835-supplementary.pdf]
